# Centenaries of Veterinary Schools

**Published:** 1891-12

**Authors:** 


### CENTENARIES OF VETERINARY SCHOOLS.

### LONDON AND MILAN.

Two great Veterinary schools have just completed their century of existence, and as is customary upon such occasions have called upon their friends to aid them in celebrating the events. We, Americans, as descendents of the Anglo-Saxon race, with affiliations with the English, and with many M. R. C. V. Ss. among our leading Veterinarians, and prominent in the Veterinary history of this country, naturally take a warmer personal interest in the centenary of the Royal Veterinary College (London), than we do in the more distant School of the Latin race at Milan. But if we put to one side our egotistical race pride and view the matter from the standpoint of Veterinary Scientists, should we do so. When we look at the gatherings at the two centenaries we find that our English School awakened but a local interest, for a day, although honored by the presence of the highest member of Royalty of the land, His Royal Highness the Prince of Wales, while that at Milan called for a congress of several days of the great Scientists of Europe, who came to honor the work of the century, which had done so much to advance, not only the Veterinary profession, but medical science at large.

The Centenary celebration of the Royal Veterinary College, London, was made the occasion of opening the new wing of the institution, Field-Marshal His Royal Highness the Duke of Cambridge, Commander-in-Chief, and President of the College, presided. He was supported by His Royal Highness the Prince of Wales, the Vice-Presidents and Governors of the Institution, representatives of the Royal Agricultural Society, and celebrities in Agriculture, Medicine and in the Veterinary profession. The President toasted the Queen and the Prince of Wales, and was responded to by the latter, who expressed his satisfaction at the results obtained and his interest in an institution which had done so much for agriculture and the army. He concluded with the following toast : " Success and continued prosperity to the institu-
"tion. It affords me the greatest pleasure to couple with it the
" name of your principal, Professor Brown, whose long professional
" career at the college and his connection with the Government
" Veterinary Department are well known to you all, and whose
" services were rewarded by the Queen on the occasion of her
" Jubilee by the Companionship of the Order of the Bath."

Professor Brown responded calling attention to the advanced scientific and social status of the Veterinarian.

A sketch of the founding of the Royal College and its history during the first quarter century of its existence, will be interesting.

